# Boost Your Brain Health: A Randomized, Controlled Trial Comparing 3 Online, Multidomain Dementia Risk Reduction Interventions

**DOI:** 10.1002/alz70860_097460

**Published:** 2025-12-23

**Authors:** Deborah E Barnes, Patricia Turo, Coles M Hoffmann, Dennis Boyle, Margaret A Chesney, Henry Brodaty, Rebecca L Sudore, Paul Tang, Linda L Chao, Matthew J Miller, Cynthia Benjamin

**Affiliations:** ^1^ University of California, San Francisco, San Francisco, CA, USA; ^2^ Linus Health, Boston, MA, USA; ^3^ apree health, Seattle, WA, USA; ^4^ IDEO, San Francisco, CA, USA; ^5^ Centre for Healthy Brain Ageing (CHeBA), UNSW Sydney, Sydney, NSW, Australia; ^6^ San Francisco VA Health Care System, San Francisco, CA, USA; ^7^ Stanford University, Palo Alto, CA, USA; ^8^ San Francisco Veterans Affairs Medical Center, San Francisco, CA, USA; ^9^ Together Senior Health, New York, NY, USA

## Abstract

**Background:**

Nearly 1 in 3 older adults are living with mild cognitive impairment (MCI) or subjective cognitive decline (SCD), which places them at increased risk for Alzheimer's disease and related dementias (ADRD). Although multi‐domain interventions may reduce dementia risk, most are time‐intensive and difficult to scale.

**Method:**

We performed a randomized, controlled trial (RCT) comparing 3 brief, online, multi‐domain interventions: 1) Brain Health Academy (BHA: asynchronous educational videos, 30 hours over 3 months); 2) Brain Health Together (BHT: synchronous group education and group mind‐body movement classes + individual brain health coaching, 30 hours over 3 months); 3) Brain Health Together Plus (BHT+: same format as BHT, 45 hours over 6 months). Eligibility criteria were age 55 years or older, English fluency, reside in U.S., self‐report MCI or SCD, and 2 or more dementia risk factors. A blinded assessor measured outcomes at 0, 3, and 6 months (primary: cognitive function [Creyos composite score] and dementia risk [modified Australian National University Alzheimer's Disease Risk Index]; secondary: validated measures of individual risk domains). Net Promoter Scores (NPS) assessed intervention satisfaction (range: ‐100 to 100, higher=better). Linear mixed models estimated standardized effect sizes (ES) for differences between groups and over time.

**Results:**

Of 760 individuals screened, 165 (18%) were eligible, consented, and randomized (55/group). Enrolled participants had a mean (SD) age of 70 (8) years; 74% were female and 28% were non‐White or Hispanic. Groups did not differ significantly at baseline. We observed significant main effects of time for both primary outcomes and most secondary outcomes, suggesting general improvement over time regardless of treatment group (ES range: 0.21 to 0.41). Loneliness declined significantly more with BHT (ES=‐0.34) and BHT+ (ES=‐0.55) than BHA (ES=‐0.01) (*p* = 0.01). NPS scores were BHA (8), BHT (26), and BHT+ (57).

**Conclusion:**

Brief, online interventions may help older adults with MCI/SCD improve cognitive function and reduce dementia risk. Synchronous programs that support participant interaction may be needed to improve loneliness. Satisfaction was highest with the longer, synchronous program. Additional studies are needed to determine whether these benefits are maintained over time.

